# Quantifying evidence toward pathogenicity for rare phenotypes: The case of succinate dehydrogenase genes, *SDHB* and *SDHD*

**DOI:** 10.1016/j.gim.2021.08.004

**Published:** 2022-01

**Authors:** Alice Garrett, Chey Loveday, Laura King, Samantha Butler, Rachel Robinson, Carrie Horton, Amal Yussuf, Subin Choi, Beth Torr, Miranda Durkie, George J. Burghel, James Drummond, Ian Berry, Andrew Wallace, Alison Callaway, Diana Eccles, Marc Tischkowitz, Katrina Tatton-Brown, Katie Snape, Terri McVeigh, Louise Izatt, Emma R. Woodward, Nelly Burnichon, Anne-Paule Gimenez-Roqueplo, Francesco Mazzarotto, Nicola Whiffin, James Ware, Helen Hanson, Tina Pesaran, Holly LaDuca, Alexandre Buffet, Eamonn R. Maher, Clare Turnbull, Cancer Variant Interpretation Group UK (CanVIG-UK)

**Affiliations:** 1 Division of Genetics and Epidemiology, The Institute of Cancer Research, Sutton, United Kingdom; 2 Central and South Genomic Laboratory Hub, Birmingham Women’s and Children’s NHS Foundation Trust, Birmingham, United Kingdom; 3 North East and Yorkshire Genomic Laboratory Hub, Central Lab, The Leeds Teaching Hospitals NHS Trust, Leeds, United Kingdom; 4 Ambry Genetics, Aliso Viejo, CA; 5 North East and Yorkshire Genomic Laboratory Hub, Sheffield Children’s NHS Foundation Trust, Sheffield, United Kingdom; 6 The Manchester Centre for Genomic Medicine and North West Genomic Laboratory Hub, Manchester University NHS Foundation Trust, Manchester, United Kingdom; 7 East Genomic Laboratory Hub, Cambridge University Hospitals Genomic Laboratory, Cambridge University Hospitals, Cambridge, United Kingdom; 8 Central and South Genomics Laboratory Hub, Wessex Regional Genetics Laboratory, Salisbury Hospital NHS Foundation Trust, Salisbury District Hospital, Salisbury, United Kingdom; 9 Cancer Sciences, Faculty of Medicine, University of Southampton, Southampton, United Kingdom; 10 Human Genetics and Genomic Medicine, Faculty of Medicine, University of Southampton, Southampton, United Kingdom; 11 Department of Medical Genetics, University of Cambridge and Cambridge University Hospitals NHS Foundation Trust, Cambridge, United Kingdom; 12 East Anglian Medical Genetics Unit, Cambridge University Hospitals NHS Trust, Cambridge, United Kingdom; 13 St. George’s University, London, United Kingdom; 14 Department of Clinical Genetics, St. George's University Hospitals NHS Foundation Trust, London, United Kingdom; 15 Cancer Genetics Unit, Royal Marsden NHS Foundation Trust, London, United Kingdom; 16 Clinical Genetics, Guy’s and St Thomas’ NHS Foundation Trust, London, United Kingdom; 17 Manchester Centre for Genomic Medicine, Manchester Academic Health Sciences Centre (MAHSC), Manchester University NHS Foundation Trust, Manchester, United Kingdom; 18 Division of Evolution and Genomic Sciences, School of Biological Sciences, Manchester Academic Health Sciences Centre (MAHSC), University of Manchester, Manchester, United Kingdom; 19 University of Paris, PARCC, INSERM, Equipe Labellisée par la Ligue contre le Cancer, Paris, France; 20 Genetics Department, Assistance Publique-Hôpitaux de Paris, Hôpital Européen Georges Pompidou, Paris, France; 21 The Wellcome Centre for Human Genetics, University of Oxford, Oxford, United Kingdom; 22 The Centre for Personalised Medicine, St Anne’s College, University of Oxford, Oxford, United Kingdom; 23 National Heart and Lung Institute and MRC London Institute of Medical Sciences, Imperial College London, London, United Kingdom; 24 Royal Brompton and Harefield Hospitals, London, United Kingdom

**Keywords:** Cancer, Germline, SDHB, SDHD, Variant interpretation

## Abstract

**Purpose:**

The weight of the evidence to attach to observation of a novel rare missense variant in *SDHB* or *SDHD* in individuals with the rare neuroendocrine tumors, pheochromocytomas and paragangliomas (PCC/PGL), is uncertain.

**Methods:**

We compared the frequency of *SDHB* and *SDHD* very rare missense variants (VRMVs) in 6328 and 5847 cases of PCC/PGL, respectively, with that of population controls to generate a pan-gene VRMV likelihood ratio (LR). Via windowing analysis, we measured regional enrichments of VRMVs to calculate the domain-specific VRMV-LR (DS-VRMV-LR). We also calculated subphenotypic LRs for variant pathogenicity for various clinical, histologic, and molecular features.

**Results:**

We estimated the pan-gene VRMV-LR to be 76.2 (54.8-105.9) for *SDHB* and 14.8 (8.7-25.0) for *SDHD*. Clustering analysis revealed an *SDHB* enriched region (ɑɑ 177-260, *P* = .001) for which the DS-VRMV-LR was 127.2 (64.9-249.4) and an *SDHD* enriched region (ɑɑ 70-114, *P* = .000003) for which the DS-VRMV-LR was 33.9 (14.8-77.8). Subphenotypic LRs exceeded 6 for invasive disease (*SDHB*), head-and-neck disease (*SDHD*), multiple tumors (*SDHD*), family history of PCC/PGL, loss of *SDHB* staining on immunohistochemistry, and succinate-to-fumarate ratio >97 (*SDHB*, *SDHD*).

**Conclusion:**

Using methodology generalizable to other gene-phenotype dyads, the LRs relating to rarity and phenotypic specificity for a single observation in PCC/PGL of a *SDHB/SDHD* VRMV can afford substantial evidence toward pathogenicity.

## Introduction

Clinical genomic analysis is typically undertaken with the aim of identifying an underlying monogenic cause in a patient with suggestive clinical features. For any genomic variant identified, a variety of evidence types are integrated to assess the likelihood of the variant being pathogenic. In 2015, the American College of Medical Genetics (ACMG) and the Association of Molecular Pathology (AMP) published a framework prescribing how these disparate evidence elements should be combined by diagnostic laboratories for classification of a newly identified genomic variant.^1^ They defined 4 strengths for the evidence elements, namely supporting, moderate, strong, and very strong, which could be combined in the classification categories of pathogenic, likely pathogenic, likely benign, and benign. However, inclusion and strength of evidence elements often differ between diagnostic laboratories and produce discrepant classifications.^2^

Pheochromocytomas and paragangliomas (PCC/PGL) are neuroendocrine tumors of the adrenal medulla and autonomic nervous system with an estimated frequency of 1 in 4000 and 1 in 16,000 respectively.^3^, ^4^, ^5^, ^6^ Head-and-neck paragangliomas (eg, chemodectoma, glomus jugulare) are derived from parasympathetic ganglia. Inherited predisposition to PCC/PGL is associated with constitutional pathogenic variants (PVs) in >15 genes, including *SDHA, SDHAF2, SDHB, SDHC, SDHD, VHL, FH, MAX, TMEM127, RET, MEN1,* and *NF1*.^4^^,^^7^ Among the Mendelian PCC/PGL cases, the most sizable contribution is from PVs in *SDHB* followed by *SDHD*.^4^^,^^8^ Associations with subphenotypes of head-and-neck paragangliomas, namely multiple, familial, and/or young-onset disease, have been reported with underlying germline PVs in *SDHA, SDHB, SDHC* or *SDHD (SDHx)* and with metastatic disease for *SDHB* PVs.^9^ The SDH proteins together form the succinate dehydrogenase enzymatic complex or mitochondrial complex II, disruption of which by PVs of any of the SDHx components may cause loss of SDHB expression in tumor material.^10^ The SDH succinate-to-fumarate ratio (SSFR) in the tumor has also been associated with underlying PVs in *SDHx*.^11^ Other tumors have been associated with PVs in *SDHx* but with much lower relative risks; these include wild-type gastrointestinal stromal tumors, SDH-deficient renal cell carcinoma, and pituitary adenomas.^10^ In the case of *SDHD, MAX,* and *SDHAF2*, disease is typically only manifested when PVs are transmitted paternally.^4^^,^^10^ For the other *SDHx* genes, the pattern of disease transmission follows the normal autosomal dominant model of inheritance.

As per the classical Knudson 2-hit model of loss-of-function, protein-truncating variants in *SDHB/SDHD* are typically pathogenic.^9^ Interpretation and classification of missense variants is more challenging. On encountering a patient with PCC/PGL and a rare missense variant in *SDHx*, evidence of pathogenicity could be inferred from (1) the very observation in an individual with the relevant rare PCC/PGL phenotype of a rare variant in an associated gene (PP2 in the ACMG/AMP framework), (2) location of that variant within a sub-region of the gene particularly associated with pathogenicity (PM1), and (3) subphenotypic features particularly associated with PVs in the *SDHx* genes, eg, invasive disease or loss of SDHB staining on immunohistochemistry (IHC) (PP4). We demonstrate generalizable quantitative approaches and requisite data sets from which likelihood ratios (LRs) can be calculated for each of these elements using the genes *SDHB/SDHD*, the phenotype PCC/PGL, and missense variation as our exemplar gene/phenotype/variant-class paradigm.^12^

## Materials and Methods

### Assembly of group clinical and laboratory experts for the gene-phenotype paradigm

Via our national United Kingdom multidisciplinary network Cancer Variant Interpretation Group UK (CanVIG-UK), we identified from the 23 United Kingdom genetics centers the lead diagnostic laboratory scientists, clinical geneticists, and endocrinologists for PCC/PGL to assemble the CanVIG-UK *SDHx* expert group, who guided sourcing of case data and focused survey of the literature.^13^

### Assembly of case variant data

For the case control analyses, we were able to identify only 1 data series providing the frequency for individual *SDHB*/*SDHD* variants, fully stratified by ethnicity, ascertained from a full *SDHx* gene analysis in a PCC/PGL series unselected for subphenotypes, which comprised 179 cases of PCC/PGL recruited to The Cancer Genome Atlas data set.^14^ We obtained summary-level per-variant frequencies for 4 additional PCC/PGL series from clinical testing; these series were all of predominantly White (Western European) ethnicity, but detailed/individual-level ethnicity data were unavailable.

The Birmingham and Leeds data sets comprised per-variant summary results from unrelated probands with PCC/PGL referred from United Kingdom clinical genetics and endocrinology centers to West Midlands Regional Genomic Laboratory Hub (2000-2020) and Yorkshire and North East Genomic Laboratory Hub (2015-2020), respectively, comprising clinical testing (single gene/gene panel including dosage analysis) of *SDHB* and *SDHD* for 3044 and 2565 patients (Birmingham) and 215 and 215 patients (Leeds), respectively. The Ambry data set comprised per-variant summary results from clinical testing for SDHx undertaken at Ambry Genetics of 1338 PCC/PGL cases referred from the US clinical genetics and endocrinology centers from 2012-2020. The French data set comprised per-variant summary results from single gene/gene panel testing of *SDHB* and *SDHD* for 1552 and 1550 patients, respectively, French PCC/PGL cases accrued 2001-2010, as previously described by Buffet et al.^8^ In total, 6328 and 5847 unrelated PCC/PGL probands were available for analysis for *SDHB* and *SDHD,* respectively. For *SDHB*, we identified in total 308 PVs predicted to truncate the protein and 315 missense variants classified in ClinVar as (likely) pathogenic. For *SDHD*, there were 155 protein-truncating PVs and 139 (likely) pathogenic missense variants (of which 116 were c.242C>T p.Pro81Leu). These classifications for missense variants are based on ClinVar (≥1 star, pathogenic/likely pathogenic) and for truncating variants on classification using ACMG criteria performed by a diagnostic clinical scientist.

For subphenotype analyses, we were able to access individual-level clinical phenotype data for a subset of 709 of the Birmingham probands, including (1) tumor location (head-and-neck/thoraco-abdominal), (2) tumor behavior (invasive/noninvasive), (3) tumor number (multiple/single), (4) family history (familial/isolated), and (5) age at diagnosis, as previously described by Andrews et al^9^ and Ricketts et al.^15^ Data on the relevant molecular subphenotypes, namely *SDHB* IHC and SSFR, were unavailable for any of our case series and thus were instead derived from the literature. We identified suitable IHC data, stratified by *SDHx* variant type, generated by Van Nederveen et al^16^ for 175 PCC/PGL cases with known germline *SDHx* status (retrospective series) and 45 PCC/PGL cases in whom *SDHx* germline testing was performed subsequently (prospective series). Two different commercial primary antibodies against *SDHB* (mouse monoclonal clone 21A11 and rabbit polyclonal HPA00286) were used to perform IHC.^16^ We identified suitable SSFR data, stratified by SDHx variant type, generated by Richter et al^11^ for 210 PCC/PGL cases (69 with PVs, 14 with variants of uncertain significance, and 127 with wild-type *SDHx*). Metabolites were measured using liquid chromatography-mass spectrometry, and variant classification was conducted according to ACMG/AMP guidelines.^1^^,^^11^

### Assembly of control data series

For the control comparison group, we made use of the publicly available gnomAD v2.1.1 (noncancer) data set: exome data from 118,479 individuals recruited via studies of common complex diseases, such as hypertension and type 2 diabetes (from which cancer-related series were excluded). The gnomAD v2.1.1 data set comprised 51,377 non-Finnish Europeans (NFEs), 10,816 Finnish Europeans, 17,130 Latino/African-Americans, 15,263 South Asians, 7451 Africans/African-Americans, 8846 East Asians, 4786 Ashkenazi Jewish, and 2810 others.^17^ To extend representation of rarer ethnicities, we also utilized the 1000 Genomes Project (1000GP) phase 3 data comprising 2504 individuals from 26 subpopulations, recognizing that some overlap between the 1000GP and gnomAD populations is reported.^17^, ^18^

### Calculation of predicted maximum tolerated allele frequency

We calculated a predicted maximum tolerated allele frequency (MTAF_pred_) for pathogenicity for a newly identified missense variant in PCC/PGL for each of *SDHB* and *SDHD* on the basis of the methods described by Whiffin et al^19^ as follows: MTAF_pred_ = disease prevalence × maximum allelic contribution × 1/penetrance, where maximum allelic contribution = genetic heterogeneity × allelic heterogeneity. MTAF_pred_ represents the estimated allele frequency in the population above which a newly identified very rare missense variant (VRMV) is not plausibly pathogenic. We sought guidance from the CanVIG-UK *SDH* expert group to ensure best estimation of the constituent parameters underpinning the MTAF_pred_ estimation.^19^

#### Disease prevalence

Because PCC/PGL is typically a time-limited condition resolved by surgery, we used lifetime risk to approximate the disease prevalence for this analysis. Estimates of the frequency in the population of PCC/PGL vary widely.^3^^,^^20^^,^^21^ For example, the estimated PCC/PGL incidence in the Netherlands was 0.04 to 0.21 per 100,000 person-years (equating to an approximate lifetime risk of ∼1 in 6000 to 1 in 31,000), whereas the estimated PCC/PGL incidence in the United States was 500 to 1600 cases per year (equating to an approximate lifetime risk of ∼1 in 2500 to 1 in 8000).^20^^,^^21^ We used a widely-cited cancer registry–derived estimate of lifetime risk for pheochromocytomas from Pacak et al^3^ of 1 in 4500 with a frequency of paraganglioma estimated to be 4-fold less common (1 in 18,000), totaling a combined lifetime incidence of 1 in 3600.

#### Penetrance

We used estimates of penetrance from the study by Andrews et al,^9^ which comprised prospective follow-ups of 371 and 67 unaffected *SDHB* and *SDHD* PV-positive nonprobands, respectively, ascertained on account of an affected index case in the family, the largest series we could identify. Penetrance to age 60 and 80 for nonprobands was estimated for *SDHB* to be 22% and 39%, respectively. For *SDHD*, penetrance in nonprobands for paternally-inherited *SDHD* PVs was estimated to be 50% to age 60.^9^ Applying to *SDHD* the proportionate age-related penetrance of *SDHB*, we thus predicted a penetrance to age 80 for paternally-inherited PVs of 88.6%. At the population-level, assuming absence of sex-selection in transmission of pathogenic *SDHD* alleles, we thus predicted an overall penetrance for *SDHD* to age 80 of approximately 44%.

#### Genetic heterogeneity

We used data from our amalgamated series to estimate genetic heterogeneity. The frequency in our PCC/PGL cases of missense (likely pathogenic/PVs) was 315 of 6328 (4.9%) for *SDHB* and 139 of 5847 (2.3%) for *SDHD*.

#### Allelic heterogeneity

Because these are well-characterized genes for which extensive clinical testing has been performed, it is likely that majorly recurrent variants have been identified, and thus, we estimated conservatively that any newly identified variant of standard penetrance is unlikely to constitute >10% of the total missense PVs.^19^

Using parameter estimates for disease frequency (1 in 3600), penetrance (*SDHB*: 0.39, *SDHD*: 0.44), genetic heterogeneity for missense variants (0.049 for *SDHB*, 0.023 for *SDHD*), and allelic heterogeneity (0.1), we estimated the MTAF_pred_ to be 1.7 × 10^–6^ for *SDHB* and 7.3 × 10^–7^ for *SDHD*.^19^ Assuming a Poisson distribution, adequate coverage, and estimates based on the lower 95th confidence interval, the MTAF_pred_ (VRMV threshold) for each of *SDHB* and *SDHD* is compatible with the observation of a maximum of 1 allele in gnomAD v2.1.1(noncancer)_NFE_ (102,754 alleles or 51,377 individuals) and a maximum of 0 allele in any of the other gnomAD v2.1.1(noncancer) subpopulations or 1000GP_all_ (largest being gnomAD Latino/African-Americans at 34,260 alleles or 17,130 individuals).

As would be anticipated, some of the more common recurrent/founder PVs occur at a frequency in controls too high for inclusion as a VRMV, namely *SDHB* c.286G>A p.Gly96Ser (frequency = 2 in v2.1.1 [noncancer]_NFE_), *SDHB* c.688C>T p.Arg230Cys (frequency = 2 in v2.1.1 [non-cancer]_NFE_), *SDHB* c.725G>A p.Arg242His (frequency = 3 in v2.1.1 [non-cancer]_NFE_), and *SDHD* c.242C>T p.Pro81Leu (frequency = 4 in v2.1.1 [noncancer]_NFE_).

### Sensitivity analysis

A sensitivity analysis was undertaken in which a range of plausible parameter estimates was tested for disease frequency (1 in 2000, 1 in 3500, 1 in 5000, 1 in 10,000), penetrance (10%-50%), and allelic × genetic heterogeneity (0.001-0.006), examining the impact on MTAF_pred_ and maximum allele count in the different-sized population data sets (Supplemental Table 1).

### Generation of LRs

We generated positive LRs and confidence intervals based on the rate of the entity under study in positives (true positive rate) compared with the rate of entity under study in negatives (false positive rate), (a/a + c)/(b/b + d), where a = true positive, b = false positive, c = false negative, and d = true negative.^22^ We generated a negative LR based on the rate of absence of the entity under study in negatives (true negative rate) compared with the rate of absence of the entity under study in positives (false negative rate), (d/b + d)/(c/a + c). When 1 or more cells contained 0 counts, we universally applied to those analyses a Haldane correction (adding 0.5 to each cell): this correction dampens a signal of association toward the null and thus is inherently conservative.

### Calculation of pan-gene VRMV-LR

The pan-gene VRMV-LR (PG-VRMV-LR) was generated as the positive LR of *SDHB/SDHD* for the frequency of VRMVs in PCC/PGL cases compared with population controls. To estimate the frequency of VRMVs in *SDHB/SDHD* in the general population, we made comparisons with the largest available single-ethnicity control population, the v2.1.1 (noncancer)_NFE_ series. We also performed a modified PG-VRMV-LR estimation in which established pathogenic VRMVs observed recurrently in the case series were excluded. We defined these as variants classified in ClinVar as pathogenic/likely pathogenic and observed in more than 8 probands in our series. This rather conservative threshold, derived from visual inspection of the frequency distribution, equates to a variant present in >1 in 800 probands or constituting in our series >5% of all VRMVs.

### Calculation of domain-specific VRMV-LRs

Using the windowing method described by Walsh et al,^23^ we performed a clustering algorithm to examine agnostically regional enrichment of VRMVs reported in cases vs VRMVs reported in controls.

### Calculation of subphenotype LRs

Clinical subphenotypic data captured at ascertainment for (1) tumor location (head-and-neck/thoraco-abdominal), (2) tumor behavior (invasive/noninvasive), (3) tumor number (multiple/single), and (4) family history of PCC/PGL (familial/isolated) were used to generate positive and negative LRs. All cases with a variant of uncertain significance in any *SDHx* gene were excluded from the wild-type *SDHx* group. Age was excluded from the multivariable analysis because on visual inspection, there was a complex relationship between PV status and age, not well captured by categorical groupings. We quantified and adjusted for colinearity among subphenotypic variables using univariable and multivariable logistic regressions. For the univariable analysis, all individuals with data for that parameter were included. For the multivariable analysis, only those with complete data on all clinical subphenotypes were included.

Using the combined van Nederveen et al^16^ series, positive and negative LRs for PVs vs wild-type *SDHx*/untested samples were calculated for loss on IHC. Using the Richter et al^11^ series, the positive and negative LRs for PVs vs wild-type *SDHx* were calculated for SSFR >97 and SSFR ≤97. For all subphenotypes, data are presented for missense PVs and all PVs.

## Results

The proportion of individuals for whom a VRMV was identified was 366 of 6328 in the PCC/PGL probands, 39 of 51,377 in the *SDHB* controls, 37 of 5847 in the PCC/PGL probands, and 22 of 51,377 in the *SDHD* controls. We calculated the PG-VRMV-LR to be 76.2 (54.8-105.9) for *SDHB* and 14.8 (8.7-25.0) for *SDHD*. PG-VRMV-LRs were broadly consistent when analyzed for the 5 case series individually. These frequencies do not include recurrent founder PVs observed in controls at a frequency exceeding the MTAF_pred_ threshold, namely *SDHB* c.286G>A p.Gly96Ser (frequency of 17 in cases and 2 in v2.1.1 [non-cancer]_NFE_), *SDHB* c.688C>T p.Arg230Cys (frequency of 10 in cases and 2 in v2.1.1 [non-cancer]_NFE_), *SDHB* c.725G>A p.Arg242His (frequency of 19 in cases and 3 in v2.1.1 [non-cancer]_NFE_), and *SDHD* c.242C>T p.Pro81Leu (frequency of 116 in cases and 4 in v2.1.1 [non-cancer]_NFE_).

However, although observed at sufficiently low frequency in controls to constitute a VRMV, a number of variants were observed in multiple independent probands and are well documented in ClinVar as (likely) pathogenic. On removal of these recurrent-pathogenic-VRMVs, the frequencies reduced to 156 in 6118 probands for *SDHB* and 37 in 5847 for *SDHD*, thus downadjusting the PG-VRMV-LR to 34.6 (24.3-49.2) for *SDHB* and 14.8 (8.7-25.0) for *SDHD* (Table 1, Supplemental Tables 2 and 3).

**Table 1:** Pan-gene VRMV likelihood ratios for *SDHB* and *SDHD*

Gene		VRMVs (all)			VRMVs (Recurrent Pathogenic Founder Variants Excluded)		
		PCC/PGL	Population Controls	Positive Likelihood Ratio	PCC/PGL	Population Controls	Positive Likelihood Ratio
*SDHB*	VRMV present	366	39	76.2 (54.8-105.9)	156	38	34.5 (24.2-49.1)
	VRMV absent	5962	51,338		5962	51,338	
	**Total**	6328	51,377		6118	51,376	
*SDHD*	VRMV present	37	22	14.8 (8.7-25.0)	37	22	14.8 (8.7-25.0)
	VRMV absent	5810	51,355		5810	51,355	
	**Total**	5847	51,377		5847	51,377	

Frequency in cases of PCC/PGL and population controls (gnomAD v2.1.1 [non-cancer]_NFE_) of VRMVs in *SDHB* and *SDHD* for (1) all VRMVs and (2) VRMVs excluding recurrent founder pathogenic variants.

*NFE*, non-Finnish European; *PCC/PGL*, pheochromocytoma and paraganglioma; *VRMV*, very rare missense variant.

**Figure 1:**
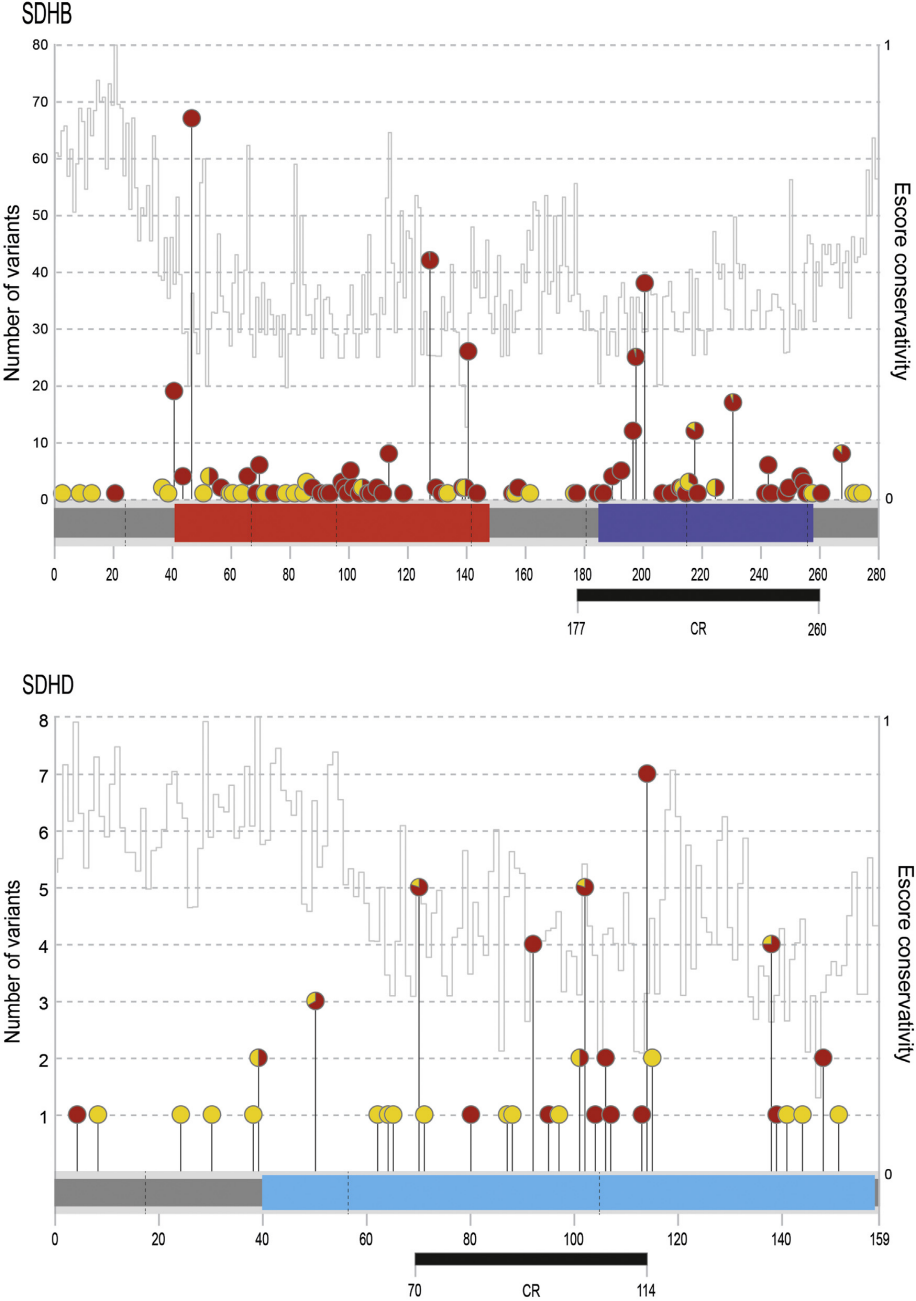
**Variant position schematic.** Lolliplot showing the position of *SDHB* and *SDHD* variants in 51,377 controls and 6328 and 5847 cases (of phaeochromocytoma and paraganglioma), respectively. Variants identified in cases are represented by red circles and those in controls are represented by yellow circles with proportional representation for variants identified in both. Exon-exon boundaries are shown with a dashed line. Protein domains are represented by colored blocks. Variant cluster regions (CR), as defined using a custom clustering algorithm (see methods), are shown as black rectangles below each protein (*P* < .004). Fer_2_3, 2Fe-2S iron-sulfur cluster binding domain (red); Fer4_17, 4Fe-4S dicluster domain (purple); CybS, succinate dehydrogenase cytochrome B small subunit (blue). Escore interspecies conservation is presented.

From the clustering analysis, we identified a region comprising 30% of the coding region of *SDHB* (ɑɑ 177-260) enriched for VRMVs in cases when compared with controls (*P* = .001). This generated domain-specific VRMV-LR (DS-VRMV-LR) of 127.2 (64.9-249.4) for variants within the region and DS-VRMV-LR of 60.9 (41.6-89.0) for those outside the region. For *SDHD,* there was also a cluster region (ɑɑ 70-114, 28% of coding region, *P* = .000003) such that DS-VRMV-LR was 33.9 (14.8-77.8) inside and 5.9 (2.6-13.0) outside of that region. Excluding the recurrent-pathogenic-VRMVs reduced the hot-DS-VRMV-LR to 59.7 (28.5-125.2) and the cold-DS-VRMV-LR to 28.2 (18.8-42.4) for *SDHB*; for *SDHD*, the DS-VRMV-LRs were unchanged (Figure 1).

Based on PV-positive vs wild-type *SDHx* case-only adjusted comparisons, invasive disease was predictive for *SDHB* missense PV status when compared with wild-type *SDHx* status (subphenotypic LR [SP-LR] = 6.5 [3.9-10.7]). Both head-and-neck disease (SP-LR = 10.6 [8.8-12.7]) and multiple tumors (SP-LR = 9.5 [5.3-17.1]) were predictive of *SDHD* missense PV status when compared with wild-type SDH wild-type. Family history of at least 1 affected first degree relative was highly predictive of missense PVs in *SDHB* (SP-LR = 18.7 [8.7-40.0]) and *SDHD* (SP-LR = 54.4 [25.6-115.5]) when compared with wild-type status (Table 2, Supplemental Tables 4 and 5). In a univariable analysis, loss of SDHB staining on IHC was strongly predictive of a PV in both *SDHB* (SP-LR = 17.9 [14.7-21.8]) and *SDHD* (SP-LR = 18.1 [16.6-19.8]) when compared with wild-type SDHx status (Table 2, Supplemental Table 6). SSFR >97 was also strongly predictive of PVs in *SDHB* (SP-LR = 108.9 [92.9-127.6]) and *SDHD* (SP-LR = 93.1 [78.3-110.8]) when compared with wild-type *SDHx* (Table 2, Supplemental Table 7). In Supplemental Table 8, some hypothetical variant scenarios are presented to illustrate a combination of these LRs under the points-based Bayesian adaptation of the ACMG variant classification framework.

**Table 2:** Case-only subphenotype analyses

Genotype		Count	LR Univariable	LR Multivariable
**Tumor location: HNPGL vs PCC/PGL w/o HN**				
*SDHB* missense PV	HNPGL	37	1.77 (1.31-2.40)	2.43 (1.80-3.29)
	PCC/PGL w/o HN	55	0.77 (0.65-0.92)	0.56 (0.47-0.67)
*SDHD* missense PV	HNPGL	31	4.26 (3.54-5.13)	10.56 (8.78-12.72)
	PCC/PGL w/o HN	1	0.04 (0.01-0.28)	0.02 (0.00-0.11)
*SDHx* wild-type	HNPGL	97		
	PCC/PGL w/o HN	330		
**Tumor behavior: invasive vs noninvasive**				
*SDHB* missense PV	Invasive	24	4.27 (2.57-7.08)	6.46 (3.89-10.72)
	Noninvasive	66	0.78 (0.69-0.89)	0.52 (0.46-0.59)
*SDHD* missense PV	Invasive	3	1.50 (0.48-4.69)	1.15 (0.37-3.60)
	Noninvasive	29	0.97 (0.86-1.08)	1.27 (1.13-1.42)
*SDHx* wild-type	Invasive	26		
	Noninvasive	390		
**Tumor number: multiple vs solitary**				
*SDHB* missense PV	Multiple	12	3.04 (1.52-6.08)	2.38 (1.19-4.77)
	Solitary	80	0.91 (0.84-0.99)	1.15 (1.06-1.25)
*SDHD* missense PV	Multiple	15	10.91 (6.09-19.55)	9.53 (5.32-17.07)
	Solitary	17	0.56 (0.40-0.77)	0.67 (0.48-0.92)
*SDHx* wild-type	Multiple	18		
	Solitary	401		
**Family history: familial vs isolated**				
*SDHB* missense PV	Familial	24	15.95 (7.45-34.16)	18.68 (8.72-40.01)
	Isolated	52	0.70 (0.60-0.81)	0.70 (0.60-0.81)
*SDHD* missense PV	Familial	16	29.93 (14.08-63.61)	54.36 (25.57-115.54)
	Isolated	11	0.42 (0.26-0.66)	0.34 (0.21-0.53)
*SDHx* wild-type	Familial	8		
	Isolated	396		
**IHC: IHC negative (abN) vs IHC positive (normal)**				
*SDHB* missense PV	IHC negative	21	17.9 (14.7-21.8)	
	IHC positive	0	0.023 (0.001-0.396)	
*SDHD* missense PV	IHC negative	53	18.1 (16.6-19.8)	
	IHC positive	0	0.010 (0.001-0.171)	
*SDHx* wild-type	IHC negative	6		
	IHC positive	112		
**SDH Succinate: Fumarate Ratio: High (>97) vs Low (≤97)**				
*SDHB* missense PV	SSFR >97	12	108.9 (92.9-127.6)	
	SSFR ≤97	2	0.14 (0.014-1.49)	
*SDHD* missense PV	SSFR >97	11	93.1 (78.3-110.8)	
	SSFR ≤97	4	0.27 (0.03-2.28)	
*SDHx* wild-type	SSFR >97	1		
	SSFR ≤97	126		

Analysis of clinical subphenotypic features in 206 *SDHB* PV-positive, 66 *SDHD* PV-positive, and 427 *SDHx* wild-type cases of PCC/PGL. Analysis of *SDHB* IHC staining in 21 *SDHB* PV-positive, 53 *SDHD* PV-positive, and 118 SDH wild-type/untested cases. Analysis of SDH SSFR) in 14 *SDHB* PV-positive, 15 *SDHD* PV-positive, and 127 SDH wild-type cases of PCC/PGL.

*HNPGL*, head-and-neck paraganglioma; *IHC*, immunohistochemistry; *PCC/PGL*, pheochromocytoma and paraganglioma; *PV*, pathogenic variant; *SSFR*, succinate-to-fumarate ratio; *w/o HN*, not in head or neck.

## Discussion

Before evolution of the ACMG/AMP framework, assignment of a variant as pathogenic was frequently based primarily on observation thereof in an individual with the correct phenotype (along with the absence on sequencing of a few hundred control chromosomes). However, this adage led to erroneous classification of many innocuous variants as pathogenic on account of (1) insufficient size of the population/control data series for confirmation of requisite rarity and/or (2) application in the context of nonspecific phenotypes such as familial breast cancer.

The notion of phenotypic specificity is not simple. For a given gene/phenotype/variant-class scenario, phenotypic specificity encompasses (1) rarity of the clinical phenotype in the general population, (2) how much of the phenotype is attributable to that gene, (3) the level of enrichment of gene variants of a particular class in that phenotype (ie, penetrance), and (4) how noisy the gene is for innocuous variants of that variant-class, and there may in addition be (5) regional variation for pathogenic vs innocuous variants of that variant-class and (6) gene-specific subphenotypic features that are particularly associated with pathogenicity.

Using *SDHB*/*SDHD*, PCC/PGL, and missense variants as our gene/phenotype/variant-class exemplar, we have demonstrated that quantitation of these LRs encompass (1) identification of a rare missense variant in an individual with the correct rare phenotype in a gene variably constrained for those variants (ACMG/AMP criterion: PP2), (2) enrichment for rare variants in cases compared with controls within specific gene regions (PM1), and (3) presence of macroscopic or molecular subphenotypic features particularly associated with germline PVs in a specific gene (PP4).

These analyses demonstrate a substantial PG-VRMV-LR for *SDHB* in particular, which is quite striking even after removal of the recurrently-reported pathogenic VRMVs. It is plausible that for other gene/phenotype/variant-class scenarios in which the gene is constrained and/or the phenotype is rare, the PG-VRMV-LR may be equivalently substantial and we may currently be underscoring evidence afforded by a single observation of a very rare variant in the relevant phenotype. Wide variation in the etiologic fraction, a similar metric, has been demonstrated for genes associated with cardiomyopathies.^24^

These analyses also exemplify the potential clinical utility of formal quantitation of LRs for subphenotypic features, in this case abnormality on IHC, high SSFR, head-and-neck disease, invasive disease, multiplex tumors, and familial disease. Although we were only able to undertake multivariable regression to adjust for collinearity between clinical subphenotypic features, collinearity between the clinical and molecular features would not be predicted a priori. Stringent technical validation would be a prerequisite for inclusion of locally-generated laboratory data: although methodology and quality assurance for IHC is well explored, there is no international reference method for SSFR, and assay thresholds may be influenced by tumor input material. The corresponding negative LR should be applied when the absence of the subphenotypic feature has high specificity for wild-type status (eg, absence of head-and-neck disease for *SDHD*). Currently, the ACMG framework lacks formal designation of a negative-phenotype evidence item.

As demonstrated by Tavtigian et al,^25^ the ACMG/AMP categorical evidence strengths can be converted to LRs (supportive LR = 2.08, moderate LR = 4.33, strong LR = 18.8, and very strong LR = 350). Thus, the 3 types of evidence items we have described can be combined together with other relevant LRs in a Bayesian framework in which the posterior probability is a function of the prior probability and the product of relevant LRs ([LR]_a_ × [LR]_b_ × [LR]_c._ × [LR]_d_ × [LR]_e…._).

Application to *SDHB*/*SDHD*–PCC/PGL cases illustrates a number of challenges and limitations in the methods presented. First, critical to the PG-VRMV-LR is conversion of the MTAF_pred_ to a filtering allele count for a given control data set. Even the largest subpopulation of the cancer-free gnomAD series (NFE) provides poor precision at low values of MTAF_pred_. Observation of 1 in 51,377 in the NFE group is consistent with the underlying frequency of 4.93 × 10^–7^ to 1.084 × 10^–4^ (95% confidence interval of binomial distribution). Accordingly, we may be overestimating the frequency of VRMVs in controls (and thus underestimating the PG-VRMV-LR). As illustrated in our sensitivity analyses (Supplemental Table 1), access to larger control series for our VRMV case control comparison will improve the precision by which we filter for MTAF_pred_.^26^

Second, limited size of non-NFE control series precludes accurate filtering of variants in non-NFE populations. Variants with a true frequency above the MTAF_pred_ may still be sufficiently rare to be absent in these modest-sized control series. Although the case series used were predominantly Western European, in the non-White minority, we may be erroneously including as VRMVs (1) variants common in ethnic groups entirely unrepresented in gnomAD or 1000GP or (2) variants absent in the respective partition of gnomAD/1000GP but at a frequency exceeding the MTAF_pred_ for the relevant ethnicity group. Erroneous inclusion of such variants as VRMVs in the case series may result in overestimation of the PG-VRMV-LRs. Furthermore, because of limited control and case data, we have had to develop parameters and apply them to the same data set. Access to additional independent data sets would allow testing of reproducibility.

Third, for most rare phenotypes, parameter estimates for population frequency, lifetime penetrance, and genetic heterogeneity vary widely and may be subject to substantial ascertainment bias. For pleomorphic syndromic phenotypes, it is only feasible to estimate MTAF_pred_ by pulling out a specific component of the syndrome and estimating the frequency, penetrance, and genetic heterogeneity for this component (eg, type 2 renal papillary cancer for the *FH* gene [hereditary leiomyomatosis and renal cell carcinoma] or medullary thyroid cancer for the *RET* gene [multiple endocrine neoplasia type 2]).^27^

Fourth, a specific case definition (ascertainment framework) is required to which the VRMV-LR is applicable. In practice, eligibility for clinical gene testing likely varies in space and time, rendering it challenging to precisely pinpoint the framework for ascertainment and case inclusion.

Fifth, the VRMV-LR metrics are based on and applicable only to observed variants that themselves are very rare (ie, are observed in the control population at frequencies below the MTAF_pred_). Variants that are disease-associated but of lower penetrance will likely occur in the population but at frequencies above the MTAF_pred_. Such variants would not have been included in the VRMV case control analyses, and the VRMV-LR metric would not be applicable to them.

Thus, although our parameter estimates were deliberately conservative and the limited size of NFE control data may have caused underestimation of PG-VRMV-LR, failure to guarantee full exclusion of VRMVs in non-NFE cases would support a conservative translation of our estimates of PG-VRMV-LRs to evidence strengths for clinical variant classification.^12^

Although we used *SDHB*/*SDHD*, PCC/PGL, and missense variation as our exemplar gene/phenotype/variant-class paradigm, the principles, requisite data sets, and methodologies illustrated here are universally applicable to any other gene/phenotype/variant-class scenario. We propose that adoption of the methodologies illustrated for other rare Mendelian cancer syndromes would improve consistency and accuracy of quantitative estimation of the rare variant/rare phenotype phenomenon (PG-VRMV-LR for PP2), of the variant location in the right hot-spot (DS-VRMV-LR for PM1), and of the quantitative evaluation of subphenotypes (SP-LR for PP4).

## Data Availability

The publicly available data analyzed are available as per the references/URLs provided. Any materials and data developed during this study will be made available upon request from the corresponding author.
